# Improvement of Quality of Life after Surgical Treatment of Patients with MRONJ: A Prospective Analysis Using the SF-12 and OHIP-14 Questionnaires

**DOI:** 10.1155/2024/4435791

**Published:** 2024-04-30

**Authors:** Georg Hoene, Nikolaus von Hahn, Denise Sievers, Lukas Schuffelen, Susanne Wolfer, Kathi Goldstein, Boris Schminke, Phillipp Brockmeyer, Philipp Kauffmann

**Affiliations:** ^1^Clinic for Oral and Maxillofacial Surgery, University Medical Center Goettingen, Robert Koch-Str. 40, 37075, Goettingen, Germany; ^2^Polyclinic for Orthodontics, University Medical Center Goettingen, Robert Koch-Str. 40, 37075, Goettingen, Germany

## Abstract

**Background:**

Medication-related osteonecrosis of the jaw (MRONJ) is a rare, serious, and debilitating disease of unknown cause that can be associated with significant health-related quality of life (HRQOL) impairment. Hematological disease is characterized by a nonhealing exposed jawbone in patients with a history of antiresorptive or antiangiogenic agent use without radiation exposure to the head or neck. *Patients and Materials and Methods*. This prospective study over the period from May 2020 to December 2021 included a representative sample consisting of 27 patients with at least stage 2 MRONJ lesions who underwent surgical rehabilitation via oral and maxillofacial surgery at the University Medical Center Göttingen, Germany. Quality of life data were collected over a 6-month postoperative period using the Health-Related QOL (SF-12) and Oral Health-Related QOL (OHIP-14) questionnaires.

**Results:**

A total of 27 patients considered in the study had a total of 42 MRONJ lesions, corresponding to a mean of 1.56 necroses per patient. MRONJ lesions were downstaged in 85% of the patients. HRQOL was evaluated with the SF-12 questionnaire. Significant improvements were found in six of the eight categories (General Health (*p*  < 0.001), Bodily Pain (*p* < 0.001), Mental Health (*p* < 0.001), Vitality (*p*  < 0.001), Role-Emotional (*p*=0.028), and Social Functioning (*p*=0.031)). The OHRQOL score also improved significantly after surgical intervention (*p* < 0.001).

**Conclusion:**

With completed surgical therapy, improvements in HRQOL and OHRQOL are measurable.

## 1. Introduction

The American Association of Oral and Maxillofacial Surgeons (AAOMS) has implemented the term “antiresorptiva-related osteonecrosis of the jaw” (ARONJ). Bisphosphonates and denosumab are antiresorptive drugs that have become indispensable in the treatment of cancer, osteoporosis, Paget's disease, and other bone diseases. As pharmacology continues to advance, particularly in the realm of biology, a recent literature review showed a diverse array of medications, such as tyrosine kinase inhibitors, monoclonal antibodies, mammalian target of rapamycin inhibitors, radiopharmaceuticals, selective estrogen receptor modulators, and immunosuppressants, linked to the development of jaw necrosis alongside established antiresorptive agents [1]. The term ARONJ was changed to the term “medication-related osteonecrosis of the jaw” (MRONJ) [2]. The incidence of NAFLD varies in the literature, ranging from 0.4% to 28% [3–5].

MRONJ is defined on the basis of all three of the following criteria: first, current or past therapy with an antiresorptive or antiangiogenic drug; second, bone in the maxillofacial region that has been exposed for more than 8 weeks; and third, no current or past radiotherapy in the head and neck region. The duration and mode of the administration of antiresorptive medication play decisive roles in the risk of MRONJ, which increases with intravenous administration and with longer therapy duration.

According to the AAOMS, MRONJ can be divided into five stages (at risk, stage 0–3). From stage 1 onward, there is an intraorally exposed and necrotic jawbone. The patients are also symptomatic. Radiological abnormalities are observed. In stage 2, there is evidence of pain and/or infection. Stage 3 is associated with possible pathological fractures or extraoral fistulas [2]. In this study, which included only patients with stage 2 or 3 MRONJ, all patients underwent surgical treatment that involved complete removal of the necrotic bone while sparing the surrounding teeth, soft tissue, and nerves. Every classification system aims to make data comparable. We consider the current AAOMS scale as the gold standard. Nevertheless, there are minor weaknesses in the classification. The boundaries between, for example, stage 2 and 3, are partially difficult to distinguish. There are no threshold values. The extent of necrosis in the bone is not captured, and the presence of symptoms or no symptoms is not clearly differentiated [6]. Antibiotic shielding was implemented during therapy [7–10].

The presence of MRONJ can lead to a significant reduction in quality of life (QOL) during its course [11, 12]. The aim of this study was to investigate QOL by using two questionnaires after surgical therapy. The Oral Health Impact Profile-14 (OHIP-14) questionnaire consists of questions about the psychological and psychosocial restrictions in the oral cavity used to define oral health-related quality of life (OHRQOL) [13, 14]. To make health-related quality of life (HRQOL) overall measurable, the SF-12 questionnaire was used; it includes questions about the general health status of the patients. The short version used in this study, the SF-12 (short form-12) questionnaire, comprises 12 questions and achieves comparable results to the version of the original questionnaire with 36 questions [15].

The purpose of this research was to investigate possible changes in the HRQOL and OHRQOL of surgically treated patients with MRONJ at different observation time points from preoperative to 6 months postoperative.

## 2. Materials and Methods

This prospective analysis was performed in 27 patients with MRONJ. The sample size of 27 was determined with *G* ^*∗*^Power (v.3.1.9.2; University of Düsseldorf, Düsseldorf, Germany) by applying a significance level of 0.05, a power of 0.8, and an estimated large effect size of 0.5.

The study included patients who presented to the Clinic for Oral and Maxillofacial Surgery at the University Medical Center Göttingen over a period from May 2020 to December 2021. The study was conducted in accordance with the guidelines of the Declaration of Helsinki and was reviewed and approved by the local ethics committee (Vote No. 7/8/20). A total of 27 patients aged 40–89 years were included in the study; 14 were female, and 13 were male.

Patients were only included in the study under certain inclusion criteria [3, 9]: an exposed jawbone for at least 8 weeks, past or current antiresorptive therapy, and no past or current head and neck radiotherapy. The inclusion criteria also included patient age older than 18 years, the presence of at least stage 2 MRONJ, and subsequent recommended surgical therapy. Furthermore, patients who developed recurrence or a new lesion of MRONJ after having completed the survey were not included a second time in the study. Patient recruitment was carried out in a consecutive manner throughout the duration of the study. Therapeutic success was defined as stage 0 at the end of the observation period of 6 months.

For clinical baseline characteristic evaluation, the main diagnosis for which an antiresorptive drug was taken, including the presence of metastasis, was recorded. In addition, the medication used, type of application, dose, duration of previous use of the administered antiresorptive agent, and any changes in medication were recorded to determine the risk profile for the development of MRONJ. The patients' treatment indication and the corresponding drug were determined by external practitioners based on their underlying medical condition. The investigators did not have a role in determining the treatment indication for the patients with the specific medication [6]. Furthermore, the location and stage of MRONJ according to the AAOMS were noted preoperatively at inclusion in the study. After surgical therapy, the healing process was documented by restaging the operated bone lesion after three and 6 months.

At five fixed points during treatment, the patients were asked about their HRQOL and OHRQOL, as achieved using two validated questionnaires, the SF-12 and the OHIP-14.

The first interview was conducted when the patients first attended consultation. In addition, necessary patient-related data were collected at this appointment, therapy options were discussed, and consent for the study was signed if all inclusion criteria were met. The second interview was always conducted 1 week after surgery. The third interview was conducted at 4 weeks, the fourth interview at 12 weeks, and the fifth interview at 6 months after surgery.

The validated SF-12 questionnaire contains 12 questions on the general health status of patients. There are questions about physical and mental limitations, acute or persistent pain, and mental satisfaction. The answer options are the same for lower-level questions but vary depending on the higher-level question. Patients were encouraged to answer the questions based on their physical and mental state over the 4 weeks before the interview, which partly overlapped with the chosen intervals of the interviews. The evaluation of this questionnaire followed a fixed scheme [15]. The remaining eight categories are shown in Table 1 with the corresponding question numbers.

The OHIP-14 questionnaire, which has also been validated, contains 14 questions on oral health-related quality of life (OHRQOL) and a supplementary question to classify patients according to the prosthetic care of their dentition. The questions address limitations in pronunciation, taste, type and satisfaction of food, appearance to others, and current pain. All variables are summarized via absolute and relative frequencies, means ± standard deviations (SDs), and medians (minimums and maximums), as appropriate. The patient-related data and the results of the questionnaires were collected and are presented graphically using the program Microsoft Excel (version 16.68). The results of the questionnaires were evaluated using SPSS Statistics (version 28.0). Descriptive analyses were carried out for the entire patient cohort as well as for specific subgroups. To validate the significance of the results, the Friedman test was used for connected samples, and the Cochran *Q* test and Cronbach's alpha test were applied. The significance level was set to alpha = 5% for all statistical tests.

## 3. Results

The baseline data of the patients, including diagnosis, age distribution, and presence of bone metastases or nicotine use, are shown in Table 2.

The 27 patients were treated with four different antiresorptive drugs based on their underlying general disease status. The majority of patients (59.3%) were receiving zolendronic acid at the time of MRONJ diagnosis. In total, nine patients received denosumab when MRONJ occurred, which corresponds to 33.3% of the patients. An overview of the medications used and how they were taken is shown in Table 3.

When analyzing the duration of medication use, the median duration between the first administration of the antiresorptive agent and the time of surgery was 61 months (5.1 years). The median age was 56 months (4.7 years), the minimum was 15 months (1.3 years), and the maximum was 200 months (16.7 years). One patient was excluded from this statistical calculation because the induction time could not be completely reconstructed. The longest interval of 200 months was based on therapy with pamidronic acid. This treatment was administered intravenously to patients with aggressive systemic mastocytosis at a dosage of 90 mg every 4 weeks beginning in 2004.

**Figure 1:**
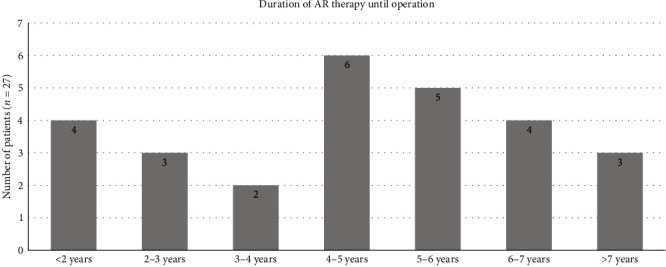
Distribution of induction times (*n* = 27).

The shortest interval was 15 months for one patient who was administered zoledronic acid intravenously at a dose of 4 mg every 4 weeks due to breast carcinoma. The correlation is shown graphically as a bar chart in Figure 1.

**Figure 2:**
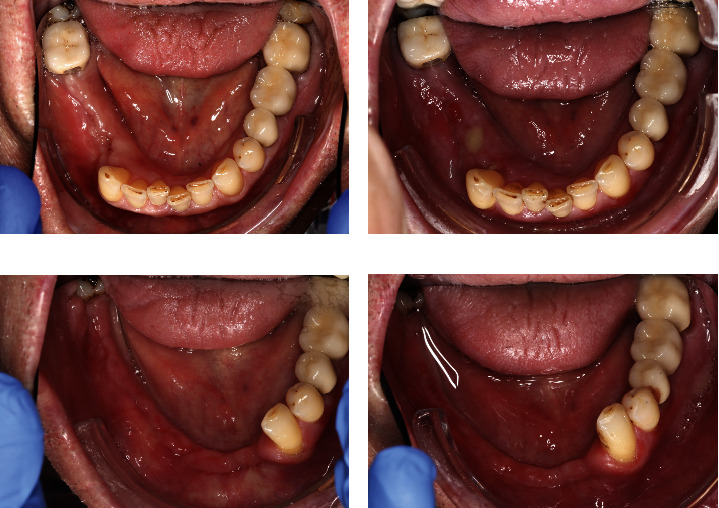
Clinical case report from the collective: (a) status at presentation after tooth extraction; (b) MRONJ stage 3 right mandible; (c) status 2 weeks postoperatively; (d) status 6 months postoperatively.

Figure 2 shows a patient from the cohort. Due to persistent pain in the right mandible after tooth extraction at a dentist, the patient was referred to our outpatient clinic. On initial admission, the status quo was as shown in Figure 2(a). With the mucosal adhesions closed, conservative therapy was applied. The patient received permanent treatment with denusomab due to osseous metastasized renal cell carcinoma. At the next checkup, the patient had MRONJ grade 3, as illustrated in Figure 2(b). Surgical intervention involving superficial debridement of the mandible; extraction of teeth 32, 31, 41, 42, 43, and 47 (*US 23*, *24*, *25*, *26*, *27*, *31*); and mucoperiosteal flap mobilization for wound closure were performed. Two weeks after the surgical intervention, the wound had healed, as shown in Figure 2(c).  Figure 2(d) shows the status at the follow-up appointment after 6 months.

A total of 27 patients considered in the study had a total of 42 MRONJ lesions, corresponding to a mean of 1.56 necroses per patient. Necrotic lesions in the same patient were considered separate if the clinically visible necrotic bone portions through the mucosa were clearly distinguishable from each other and were located in other regions of the jaw. Of the 42 lesions, 16 were located in the maxilla and 26 in the mandible, representing a ratio of 38.1%–61.9%.

A total of 15 patients were affected by lesions in the mandible, whereas only seven patients were affected by lesions in the maxilla. The remaining five patients had necrotic lesions in both the maxilla and mandible.

The study design included a total of five surveys on QOL during the course of surgical therapy. At three time points during therapy, MRONJ and the surgically treated areas were graded. In patients who had several lesions at the beginning of surgical therapy, the lesion with the highest stage was scored. At baseline, 25 of the 27 patients were in stage 2, and two patients were in stage 3. Three months after surgical removal of the MRONJ, 17 patients were in stage 0, six patients were in stage 1, and four patients were in stage 2. All patients who were diagnosed with stage 2 disease again 3 months postoperatively were also diagnosed with stage 2 disease at baseline. Two patients with stage 3 disease had stage 0 or stage 1 disease after 12 weeks. At the last stage 6 months after surgery, 13 patients were in stage 0, 10 patients were in stage 1, and four patients were still or again in stage 2.

No downstaging could be achieved in these last four patients. Two of the cases had regressed to stage 0 at 3 months but then deteriorated to a new stage 2 in the following months. The other two patients had stage 2 MRONJ throughout the entire observation period despite the surgery performed.

**Figure 3:**
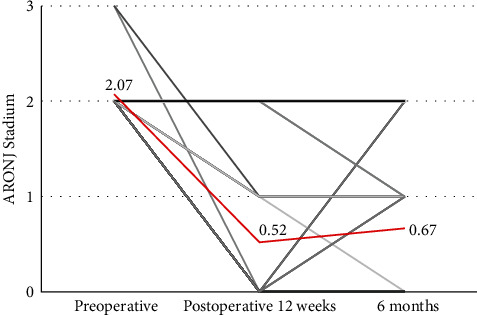
Graphical representation of the stages (*n* = 27).

Therefore, downstaging was successful in a total of 23 (85%) of 27 patients. Therapeutic success, defined as stage 0 at the end of the observation period of 6 months, however, occurred in only 13 patients (48%). Among the other 14 patients (52%), the surgically treated MRONJ was still in stage 1 (10 patients; 37%) or even in stage 2 (four patients; 15%). An overview of the progression of the disease is shown in Figure 3. The mean values are illustrated in the red graph (preoperative MV = 2.05 ± SD = 0.26; postoperative 12-week MV = 0.52 ± SD = 0.75; after 6 months, MV = 0.52 ± SD = 0.72).

HRQOL was evaluated with the SF-12 questionnaire. Nonparametric tests in the form of linked samples were conducted to validate the significance of the results of the SF-12 questionnaire. The SF-12 score was evaluated for each of the eight categories individually to determine whether the change in percentile rank and thus HRQOL during the five surveys was significant.

Six of the eight categories (General Health (GH), Physical Functioning (PF), Bodily Pain (BP), Mental Health (MH), Vitality (VT), and Social Functioning (SF)) were evaluated using Friedman's two-factor analysis of variance for ranks. The Role-Physical (RP) and Role-Emotional (RE) categories were evaluated using the Cochran *Q* test with connected samples due to the dichotomous response options of “yes” or “no.” According to both the Friedman and Cochran *Q* tests, the null hypothesis was that HRQOL does not change during therapy. Subsequently, Dunn–Bonferroni correction was performed post hoc.

This null hypothesis could be refuted with high significance in the four health domains “General Health” (*p* < 0.001), “Bodily Pain” (*p* < 0.001), “Mental Health” (*p* < 0.001), and “Vitality” (*p* < 0.001). The null hypothesis could also be rejected for the categories “Role-Emotional” (*p*=0.028) and “Social Functioning” (*p*=0.031).

Nonsignificant results were obtained for the “Physical Functioning” (*p*=0.343) and “Role-Physical” (*p*=0.582) categories. For these two physical characteristics of HRQOL, it could not be shown whether HRQOL changes or improves during therapy.

**Figure 4:**
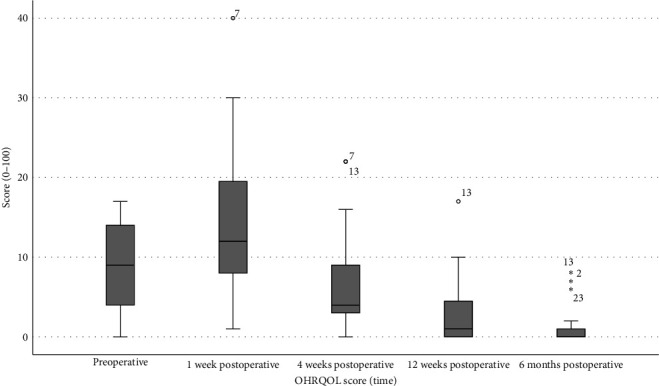
OHRQOL boxplot: The figure shows a boxplot of ORHRQOL during the study period from preoperative to 6 months postsurgery.

To test the significance of the results of the OHIP-14 questionnaire, two-factor analysis of variance according to Friedman was performed for the entire patient population. Post hoc significance values were adjusted by Bonferroni correction for multiple tests. The null hypothesis that oral health-related quality of life (OHRQOL) does not change during therapy over the five survey time points could be significantly (*p* < 0.001) refuted. According to pairwise comparisons, an increase in OHRQOL between neighboring time points of the survey was significant (*p* < 0.001). The OHRQOL score remained the same between the fourth and fifth interviews (*p*=0.132). Similarly, a nonsignificant difference was found in the number of consecutive examinations between the first and third surveys (*p*=0.439). Thus, OHRQOL was at the preoperative level or slightly above 4 weeks after surgery but improved steadily after surgery. To illustrate this relationship, a boxplot is shown in Figure 4.

## 4. Discussion

Over the last few years, the assessment of patients' quality of life and psychological well-being has become the focus of medical research [16]. The aim of this study was to prospectively assess the QOL of patients with advanced MRONJ after surgical intervention using two questionnaires. The SF-12 assessed HRQOL, and the OHIP-14 assessed OHRQOL. Surgical therapy resulted in the downstaging of MRONJ lesions in 85% of the patients. Therapeutic success, defined as stage 0 at the end of the observation period of 6 months, however, occurred in only 13 patients (48%). This has also been shown in other studies, in which surgical intervention achieved better therapeutic results than conservative therapy [17, 18]. The concept of therapeutic success is subjective and individualized for each patient. For some patients, maintaining a certain classification level following surgical intervention can be considered a personal achievement if it is associated with a decrease in clinical symptoms, a smaller defect size, which is not reflected in the AAOMS classification and an enhancement in QOL.

The present prospective study included a representative cohort. The average age of the patients was 73 years. Comparable age structures have been described in several studies [19, 20]. Regarding sex distribution, similar studies can be found [21] as can studies with more female patients [20, 22] and more male participants [11]. The main underlying diseases in this study varied. In summary, 77.8% of the patients were affected by malignant diseases, and 22.2% were affected by some form of osteoporosis. Similar findings have been reported [9].

The development of MRONJ leads to deterioration in QOL. This phenomenon has been investigated in numerous studies [22–24]. In these studies, however, QOL is usually the only secondary parameter considered alongside primary therapeutic results. Investigations of QOL or even development of acquired MRONJ during therapy have rarely been carried out. This aspect is one of the strengths of this study.

The SF-12 is an appropriate tool for assessing HRQOL reasonably [25]. The evaluation of the results revealed significant improvements in six of the eight health domains during the observation period and after surgical treatment. The domains included “General Health” (*p* < 0.001), “Bodily Pain” (*p* < 0.001), “Mental Health” (*p* < 0.001), “Vitality” (*p* < 0.001), “Role-Emotional” (*p*=0.028), and “Social Functioning” (*p*=0.031). Only the other two health domains, “Physical Functioning” (*p*=0.343) and “Role-Physical” (*p*=0.582), showed no significant differences. Capocci et al. [22] also used the SF-12 questionnaire to assess HRQOL. Based on their 30 patients, they were able to show that the scores in both the physical and mental categories of the SF-12 were lower than those in the general Italian population. However, the impact of surgical intervention on HRQOL was not investigated. Similarly, reduced general QOL was shown in the study by de Cassia Tornier et al. [24] using the EORTC QLQ-C30 (European Organization for Research and Treatment of Cancer Quality of Life Questionnaire 30). In addition, Winter et al. [26] used the SWLS questionnaire, a comparable tool, to measure HRQOL. The patients did not demonstrate any significant improvement after surgical treatment. However, they found that patients with smaller MRONJ defects had greater HRQOL.

The study with the greatest similarity to the present study thus far is that by Moll et al. [21]. With QOL as the primary factor, patients with stage 3 MRONJ were interviewed during the course of their surgical therapy. The interviews also involved two different questionnaires. One was the OHIP-14, which was also used in this study, and the other was the EORTC QLQ-H&N35 (European Organization for Research and Treatment of Cancer Quality of Life Questionnaire Head and Neck Cancer Module 35). The downstaging rate was similar to our results (81%). The evaluation of both questionnaires showed significant improvements in HRQOL and OHRQOL, as in our study [21].

To measure OHRQOL, we used the OHIP-14 in this study. According to pairwise comparisons, an increase in OHRQOL between neighboring time points of the survey was significant (*p* < 0.001). Other studies have shown that OHIP scores improve more than non-OHIP scores after surgical intervention in patients with MRONJ stage 2 disease [26, 27].

The study by Miksad et al. [11] has a design partially similar to that of our study. The authors also used the OHIP-14 questionnaire to assess OHRQOL. They found that the development of MRONJ leads to poorer OHRQOL, as indicated by an increase in total OHIP-14 scores. These problems were related to pain, food intake, interruption of food intake, irritability, insecurity related to the oral area, and decreased satisfaction with life. The comparative values in our study showed better values for OHIP evaluations. The mean score of the first survey in the present study was 8.56, which was significantly lower than the 16.53 points of Miksad et al. [11]. Studies have found bidirectional effects in the relationship between QOL and MRONJ. Specifically, low scores on Oral Health Impact Profile assessments may increase the risk of exacerbating osteonecrosis of the jaw or serve as a significant risk factor for its development [28].

This study has potential limitations. There are confounding factors that affect the validity of our results. The main limitation is the small number of patients, which was due to the low incidence of MRONJ in the general population [3–5]. This finding contrasts with the findings of the other studies mentioned above, in which patient numbers were comparable [11, 21, 22]. Despite the small sample size, we believe that the intergroup consistency is high. The Cronbach's alpha values for both the HRQOL (C_*α*_ > 0.96) and OHRQOL (C_*α*_ > 0.76) questionnaires were high and comparable to those of other studies [26, 29]. Furthermore, our studies are limited by the absence of a control group consisting of patients with advanced MRONJ who do not undergo surgical treatment, a scenario that is ethically unfeasible. We believe that the prospective design of the study is a quality characteristic of this work. In future studies, larger patient groups should be examined over longer periods of time. In our investigation, the causal relationship between the lower OHIP-14 and SF-12 scores observed in MRONJ patients and the underlying condition necessitating AR therapy remains indeterminate. Subsequent case–control studies are warranted to compare individuals with identical underlying conditions, both with and without MRONJ, in order to elucidate this association. Moreover, standardization of questionnaires for better comparability would be appropriate [30]. Likewise, the utilization of specific Patient Reported Outcome Measures (PROMs) questionnaires with established psychometric properties would enhance validity, as generic QOL measures may not sufficiently capture mood disturbances [16].

## 5. Conclusion

Patients with MRONJ grade 2 or worse benefit from surgical therapy in terms of their HRQOL and OHRQOL. MRONJ lesion downstaging was successful in 85% of our patients. After initial deterioration, significant improvements in quality of life were achieved in the majority of patients.

## Figures and Tables

**Table 1:** All health domain scales and their corresponding questions.

	Health domain scale	Question number
1	General Health (GH)	1
2	Physical Functioning (PF)	2 + 3
3	Role-Physical (RP)	4 + 5
4	Role-Emotional (RE)	6 + 7
5	Bodily Pain (BP)	8
6	Mental Health (MH)	9 + 11
7	Vitality (VT)	10
8	Social Functioning (SF)	12

**Table 2:** Baseline data.

Baseline data						
Sex	Male	Female	Total	—	—	—
	13	14	27	—	—	—
	48.10%	51.90%	—	—	—	—

Age	Mean	Median	Min.	Max.	Mean male	Mean female
	73 years	75 years	40 years	89 years	76 years	71 years

Nicotine	Smoker	Nonsmoker	—	—	—	—
	7	20	—	—	—	—
	26%	76%	—	—	—	—

Bone metastases	Yes	No	—	—	—	—
	19	8	—	—	—	—
	70.40%	29.60%	—	—	—	—

Main diagnosis	Cases	%	—	—	—	—
Breast cancer	5	18.5%	—	—	—	—
Prostate cancer	5	18.5%	—	—	—	—
Multiple myeloma	4	14.8%	—	—	—	—
Osteoporosis	4	14.8%	—	—	—	—
Renal cell carcinoma	3	11.1%	—	—	—	—
Others	6	22.2%	—	—	—	—
Total	27	100.0%	—	—	—	—

**Table 3:** Distribution of drugs and route of administration (*n* = 27).

Medication	Dose	No. of pat. (%)
Alendronate (oral)	70 mg once weekly	1 (3.7%)
Pamidronate (i.v.)	90 mg once monthly	1 (3.7%)
Zoledronate (i.v.)	4 mg once monthly	16 (59.3%)
Denosumab (s.c.)	60 mg every 6 months (7 patients) 120 mg once monthly (2 patients)	9 (33.3%)
Route of administration	—	—
Oral	—	1 (3.7%)
Subcutaneous	—	9 (33.3%)
Intravenous	—	17 (63.0%)

## Data Availability

All data of the study can be retrieved at any time upon request to the corresponding author.
